# Geographical variation in the association of child, maternal and household health interventions with under-five mortality in Burkina Faso

**DOI:** 10.1371/journal.pone.0218163

**Published:** 2019-07-01

**Authors:** Ourohiré Millogo, Jean Edouard Odilon Doamba, Ali Sié, Jürg Utzinger, Penelope Vounatsou

**Affiliations:** 1 Swiss Tropical and Public Health Institute, Basel, Switzerland; 2 University of Basel, Basel, Switzerland; 3 Centre de Recherche en Santé de Nouna, Nouna, Burkina Faso; University of Kwazulu-Natal, SOUTH AFRICA

## Abstract

**Background:**

Over the past 15 years, scaling up of cost effective interventions resulted in a remarkable decline of under-five mortality rates (U5MR) in sub-Saharan Africa. However, the reduction shows considerable heterogeneity. We estimated the association of child, maternal, and household interventions with U5MR in Burkina Faso at national and subnational levels and identified the regions with least effective interventions.

**Methods:**

Data on health-related interventions and U5MR were extracted from the Burkina Faso Demographic and Health Survey (DHS) 2010. Bayesian geostatistical proportional hazards models with a Weibull baseline hazard were fitted on the mortality outcome. Spatially varying coefficients were considered to assess the geographical variation in the association of the health interventions with U5MR. The analyses were adjusted for child, maternal, and household characteristics, as well as climatic and environmental factors.

**Findings:**

The average U5MR was as high as 128 per 1000 ranging from 81 (region of Centre-Est) to 223 (region of Sahel). At national level, DPT3 immunization and baby post-natal check within 24 hours after birth had the most important association with U5MR (hazard rates ratio (HRR) = 0.89, 95% Bayesian credible interval (BCI): 0.86–0.98 and HRR = 0.89, 95% BCI: 0.86–0.92, respectively). At sub-national level, the most effective interventions are the skilled birth attendance, and improved drinking water, followed by baby post-natal check within 24 hours after birth, vitamin A supplementation, antenatal care visit and all-antigens immunization (including BCG, Polio3, DPT3, and measles immunization). Centre-Est, Sahel, and Sud-Ouest were the regions with the highest number of effective interventions. There was no intervention that had a statistically important association with child survival in the region of Hauts Bassins.

**Interpretation:**

The geographical variation in the magnitude and statistical importance of the association between health interventions and U5MR raises the need to deliver and reinforce health interventions at a more granular level. Priority interventions are DPT3 immunization, skilled birth attendance, baby post-natal visits in the regions of Sud-Ouest, Sahel, and Hauts Bassins, respectively. Our methodology could be applied to other national surveys, as it allows an incisive, data-driven and specific decision-making approach to optimize the allocation of health interventions at subnational level.

## Introduction

Under-five mortality remains a major public health issue in sub-Saharan Africa, despite a remarkable decline during the Millennium Development Goal (MDG) era from 2000 to 2015 [1]. The under-5 mortality rate (U5MR) estimates in 2016 suggest that one in twelve children of sub-Saharan Africa did not reach their fifth birthday [2]. Pneumonia, preterm birth complications, intrapartum events, and diarrhoea constitute the main causes of under-5 deaths in sub-Saharan Africa [3]. Indirect factors related to child, maternal, family, community, and the environment are also strongly associated with under-5 mortality, and hence underlie theses direct causes [3–5]. Most of the direct and indirect causes are preventable. During the MDG era, facilitated by the commitment of donors, local governments, and other stakeholders cost-effective interventions were scaled up. The effects of the interventions show considerable spatial heterogeneity.

Since 2006, Burkina Faso scaled up child health interventions consisting of subsidy of deliveries, artemisinin-based combination therapies (ACTs), rapid diagnostic tests (RDTs) for malaria at health facility and community levels, and universal distribution of long lasting insecticidal nets (LLINs). Furthermore, new vaccines have been included in the Expanded Programme of Immunization (EPI). The U5MR declined from 146.9 per 1000 to 88.6 per 1000 between 1998 and 2015 [6]. Likewise in other Sub-Saharan Africa countries the reduction was heterogeneous between and within the different administrative regions of the country [7–11]. For example, the DHS 2010 data showed that the U5MR in the region of Sahel was four times higher than that of Centre-Est [6]. In the rural district of Nouna, the U5MR declined by more than 50% during the last 25 years, however strong disparities in mortality have been observed within the district, which are likely to be related to disparities in risk factors and coverage of health interventions [10]. Even though the coverage of child and maternal interventions has increased globally at national level, there are large regional disparities within the country. For example, household ownership of at least one LLIN was more than 90% in the regions of Plateau Central and Nord, while it was only 41% in the Centre-Nord region [6]. The breastfeeding rate within one hour after birth varied from 27% to 67%. Regional variation also exists in the skilled antenatal and birth attendance across the country. Results of the Malaria Indicator Survey (MIS) in 2014 showed that in the region of Centre and Centre-Ouest, around 2% of children received an ACT on the same or the next day after the onset of fever, while in the region of Cascades, ACT coverage was much higher (42%) [12]. Moreover, data from routine health management information system confirm the same heterogeneity observed in the MIS data [12]. The variability in the coverage of health interventions is related to external factors including deficiencies in the health system, which affect their effectiveness. In Burkina Faso, very few studies have assessed the association between health interventions and U5MR at subnational level. The few available studies assessed a subset of interventions [13].

Our aim was to assess the magnitude of association between child, maternal and household health interventions and under-five mortality at national and sub-national levels. We hypothesize that there is a geographical variation in the effects of health interventions and we aim to identify the interventions and the regions where there is a statistically important association between intervention coverage and child mortality. Our data will support decision making for delivering the most effective interventions in those regions where the highest rate were predicted.

## Methods

### Study area

Burkina Faso is a country in West Africa, with an estimated population of 18.5 millions inhabitants in 2015. Around 40.1% of the population lives below the poverty threshold and the Human Development Index (HDI) is 0.402. The population is relatively young with 21% and 54% below 5 and 18 years, respectively. The country is part of the Sudanian zone with a dry tropical climate and two seasons: a dry season from November to June characterised by a peak of respiratory diseases and a wet season from July to October with malaria as the most important communicable disease.

### Data source

U5MR and health intervention data were extracted from the Burkina Faso Demographic and Health Survey (DHS) carried out in 2010. The data were collected from a two-stage cluster design and are representative at the national level, for urban and rural areas and for the 13 administrative regions. The survey was carried out in 574 georeferenced clusters and included 17 087 women of reproductive age who provided information for 15 375 lives births in the five years before the survey.

We extracted information of selected key health interventions of the countdown to 2015 initiative with less than 15% of missing values [14]. In particular, we included the following child-related health interventions: all antigens, measles, and DPT3 immunization, vitamin A supplementation, use of long lasting insecticidal nets (LLINs), malaria treatment by any anti-malarial, exclusive breastfeeding, immediate breastfeeding after birth, and baby post-natal check. We considered maternal interventions, such as skilled birth and antenatal care, post-natal check, family planning and intermittent preventive treatment of malaria during pregnancy (IPTp). The household specific interventions included in the study were improved drinking water source and sanitation, wealth index, and ownership of LLIN. We include malaria interventions (sleeping under ITN, household ownership of ITNs and malaria treatment) in the list of child health intervention because children under-5 years old are at higher risk of morbidity and mortality in malaria endemic countries such as Burkina Faso. Data measuring coverage of health interventions were aggregated at regional level. A description of the health intervention coverage indicators used in this study is given in S1 Table. Furthermore, we extracted information on socio-demographic characteristics of mothers and children under-5years of age such as birth order, sex, and place of delivery, mother’s age at first birth, her literacy level, as well as the number of live births.

Environmental and climatic factors, such as land surface temperature (LST), vegetation indices (Enhanced Vegetation Index (EVI) and Normalized Difference Vegetation Index (NDVI)), distance to water bodies, and type of land cover were compiled from satellite sources. Day and night LST and rainfall data were averaged for year 2010. Permanent water bodies were obtained from the land cover category. Details on the source of climatic data and their spatio-temporal resolution are given in S2 Table. The shapefile of Burkina Faso was extracted from the “The Humanitarian Data Exchange” database at: https://data.humdata.org.

### Statistical methods

Bayesian geostatistical proportional hazard models with a Weibull baseline hazard were fitted on child mortality data to assess the association between child, maternal, and household health interventions and U5MR. The models were adjusted for child, maternal, socio-demographic characteristics, climatic and environmental factors. Spatial correlation was introduced by a Gaussian process, adopted on cluster-specific random effects with an exponential correlation function of the distance between survey clusters. Spatially varying regression coefficients for the interventions were used to capture the geographical variation of the association at sub-national level and they were modelled by regional random effects with a conditional autoregressive prior distribution. That is, let *s* = {*s*_1_, *s*_2_, …, *s*_*m*_}, *s*_*i*_ ∈ *D* ⊂ *R*^2^ be the set of locations at which mortality data are observed, *t*_*j*_(*s*_*i*_) be the time to death or the censoring time (in months) for child *j* at location *s*_*i*_, ***X***_***j***_(*s*_*i*_) be the vector of child, maternal, socio-demographic and climatic factors and *Z*(*s*_*i*_) be the coverage of a given intervention at location *s*_*i*_. We modeled the mortality hazard as, *h*(*t*_*j*_(*s*_*i*_)) = *h*_0_(*t*_*j*_(*s*_*i*_))exp(***β***^*T*^***X***_***j***_(*s*_*i*_) + (*a* + *w*_*q*(*i*)_)*Z*(*s*_*i*_) + *φ*(*s*_*i*_)) and assumed a Weibull baseline hazard i.e. *h*_0_(*t*_*j*_(*s*_*i*_)) = *δ*(*t*_*j*_(*s*_*i*_))^*δ*−1^ where *δ* is the shape parameter, ***β***^T^ = (*β*_1_, …, *β*_*p*_) is the vector of regression coefficients with exp(*β*_*l*_), *l* = 1, …*p*, corresponding to the hazard ratio (HR). *φ*(*s*_*i*_) is a cluster-specific random frailty which captures spatial correlation in mortality i.e. clusters in closer proximity are expected to have similar mortality hazard due to common exposures. We modeled ***φ***(*s*) = (*φ*(*s*_1_), *φ*(*s*_2_), …, *φ*(*s*_*m*_))^*T*^ by a Gaussian process, i.e. ***φ***(*s*)~*N*(0,*σ*^2^*R*), with an exponential correlation function of the distance *d*_*kl*_ between locations *s*_*k*_ and *s*_*l*_, that is *R*_*kl*_ = exp(−*d*_*kl*_*ρ*). The parameter *σ*^2^ corresponds to the variance of the spatial process and *ρ* controls the rate of correlation decay with distance. For the exponential correlation function, $\frac{-log(0.05)}{\rho }$ determines the distance at which the correlation drops to 0.05 (i.e. effective range of spatial process). The geographical variation in the association between interventions and U5MR was modelled by the spatially varying coefficients, *a* + *w*_*q*(*i*)_ where *a* quantifies the magnitude of the association at global (national) level and ***w*** = (*w*_1_, …, *w*_*Q*_)^***T***^ are the varying effects at regional (sub-national) levels *q* = 1, … *Q* with *q*(*i*) indicating the region *q* corresponding to the location *s*_*i*_. We introduced spatial dependence among the regions via a conditional autoregressive (CAR) prior for ***w***, that is $w\sim N(0,{\sigma_{q}^{2}R}_{q})$ with $R_{q}={\left(I-\gamma C\right)}^{-1}D.\sigma_{q}^{2}$ is the variance of the spatially varying intervention coefficients, *D* is a diagonal matrix with entries *D*_*kk*_ = *g*_*k*_^−1^ where *g*_*k*_ is the number of neighbors of region *k*, *γ* measures overall spatial dependence and *C* is a proximity matrix with normalized entries that is *C*_*kl*_ = *ω*_*kl*_/*g*_*k*_, *ω*_*kl*_ is 1 if region *k* neighbors *l* and 0 otherwise [15]. To complete Bayesian model formulation, we assumed inverse gamma priors for all spatial variances with known parameters, i.e. $\sigma^{2},\sigma_{q}^{2}\sim IG(2.01,1.01)$, a uniform prior distribution for *ρ*~*U*(*a*, *b*), where *a* and *b* chosen such as the effective range is within the maximum and minimum distances of the observed locations and a uniform prior for $\gamma \sim U(\lambda_{1}^{-1},\lambda_{2}^{-1})$, where *λ*_1_, *λ*_2_ are the smallest and largest eigenvalue of D-12CD12 The shape parameter was assigned an exponential prior *δ*~*exp*(0.01). Non-informative normal priors were adopted for the regression coefficients *β*_*l*_, *a*~*N*(0, 10^3^) for *l* = 1, … *p*.

Model parameters were estimated using Markov Chain Monte Carlo (MCMC) simulation. We run a two chain algorithm for 300 000 iterations with an initial burn-in of 15,000 iterations. Convergence was assessed by the Gelman and Rubin diagnostic [16].

Prior to Bayesian spatial analysis, bivariate, non-spatial, Weibull proportional hazards models were fitted to identify potential child, maternal and socio-economic confounders. Variables with p-value less than 0.15 were included in the geostatistical model.

The statistical analyses were carried out in STATA version 14 (StataCorp.; College Station, TX, USA) and OpenBUGS version 3.2.3 (Imperial College and Medical Research Council; London, UK). Maps were produced in ArcGIS version 10.2.1 (Esri Inc.; Redlands, CA) and graphs in R (R Core Team; Vienna, Austria).

### Ethical approval

We used secondary data that was made available by the MEASURE Demographic Health Survey (DHS) Program based in the United States of America. According to the survey report [6], ethical approval was obtained by the institutional review board of ICF of Calverton, Maryland, USA and the national ethics committee for health research of Burkina Faso under deliberation N°2014-7-072. The survey was anonymous. Blood samples were taken from all eligible children for whom parents or responsible adults had given their informed consent.

## Results

**Fig 1:**
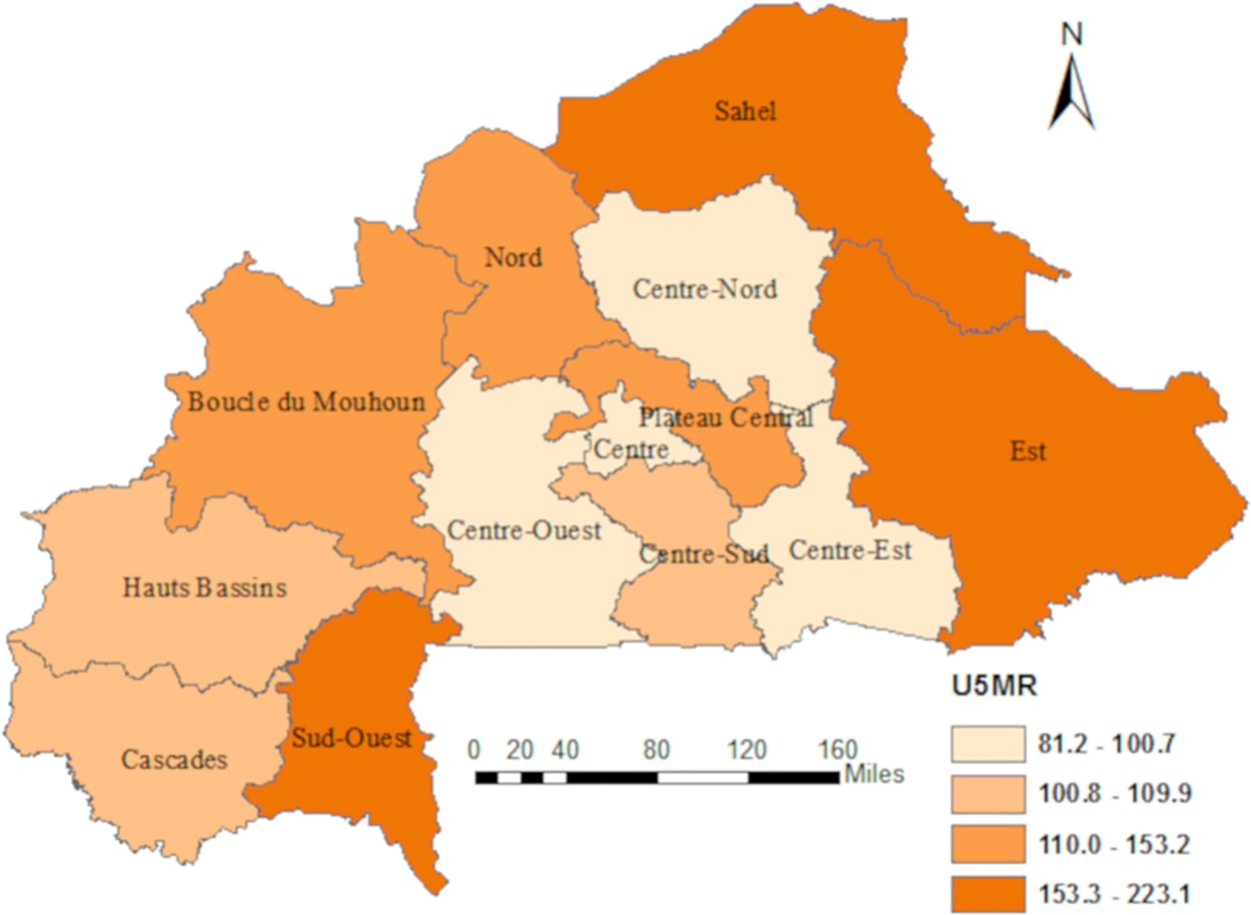
Regional distribution U5MR in Burkina Faso based on DHS 2010.

Our sample included 541 (94.3%) clusters and 13 505 (87.8%) children under the age of five years, after removing clusters with missing coordinates. In total, 1209 (9%) children died before their fifth birthday owing to an estimate for U5MR of 128 per 1000. The geographical distribution of U5MR is shown in Fig 1. The highest U5MRs were observed in the regions of Est, Sahel, and Sud-Ouest with respective U5MR of 172, 197, and 223 per 1000. The Centre-Est had the lowest rate of 81 per 1000.

Approximately two-third of the children were born in health facilities and 86% lived in rural areas. About 5% of mothers were younger than 19 years of age, two-third gave their first birth before age 19 years and 84% were not educated. Around one quarter of the respondents have more than five children and about 44% of the households were relatively poor (i.e. household asset in the first two quintiles). The socio-demographic characteristics of the sample are summarised in Table 1.

**Table 1:** Child, maternal and household characteristics and hazard rate ratios estimated by bivariate Weibull proportional hazards models.

Covariate	Percentage (%) N = 13 505	Number of death (%)	Hazard rate ratio (95%CI)	P value
**Children characteristics**				
Sex				
*Female*	49.1	566 (8.5)	1.00	
*Male*	50.9	643 (9.4)	1.12 (0.99–1.25)	0.063
Place of residence				
*Urban*	13.6	104 (5.7)	1.00	
*Rural*	86.4	1 105 (9.5)	1.71 (1.40–2.10)*	<0.001
Place of delivery				
*Health facility*	66.3	650 (7.3)	1.00	
*Home*	32.7	559 (12.3)	1.57 (1.40–1.76)*	<0.001
Birth order				
*1–5*	75.3	863 (8.5)	1.00	
*>5*	24.7	346 (10.4)	1.26 (1.11–1.43)*	<0.001
**Mothers characteristics**				
Age group (years)				
< 19	4.6	76 (12.3)	1.00	
20–35	75.3	870 (8.6)	0.48 (0.38–0.61)*	<0.001
>35	20.1	262 (9.7)	0.51 (0.40–0.69)*	<0.001
Age at first birth				
*≤19*	66.01	850 (9.5)	1.00	
*>19*	34.0	359 (7.8)	1.22 (1.07–1.39)*	0.001
Number of live birth				
*1–5*	71.0	750 (7.8)	1.00	
*>5*	29.0	459 (11.7)	1.47 (1.31–1.65)*	<0.001
Mother education level				
*Primary and above*	14.0	104 (5.5)	1.00	
*No education*	86.0	1 11 (9.5)	1.72 (1.41–2.11)*	<0.001
**Households characteristics**				
Asset index				
*Richest*	33.7	301 (6.6)	1.00	
*Middle*	22.5	280 (9.2)	1.41 (1.20–1.67)*	<0.001
*Poorer*	43.8	628 (10.6)	1.62 (1.42–1.86)*	<0.001

*: Statistically significant association (i.e.P-value<5%)

Coverage estimates of the child, maternal, and household health interventions used in the study, stratified by region are given in Table 2. The corresponding intervention coverage maps are shown in S1 and S2 Figs.

**Table 2:** U5MR and coverage of child, maternal and household health interventions stratified by region, as assessed by the Burkina Faso DHS 2010.

Health Interventions (%)	Administrative regions													
	Boucle du Mouhoun	Cascades	Centre	Centre-Est	Centre-Nord	Centre-Ouest	Centre-Sud	Est	Hauts Basins	Nord	Plateau Central	Sahel	Sud-Ouest	National level
**Child interventions**														
Use of ITNs by under-5 years old	42.6	49.8	33.1	35.9	32.0	46.6	38.2	46.0	36.2	65.7	73.5	37.3	44.7	43.4
Malaria treatment	22.5	19.2	36.1	47.9	23.1	36.8	45.7	42.2	42.0	42.0	40.5	20.1	31.7	35.7
Exclusive breastfeeding	6.7	10.1	2.4	8.8	8.6	6.8	7.3	9.0	7.3	6.0	7.3	5.2	5.9	7.0
Breastfeeding after birth within 24 h	36.4	45.6	55.3	26.6	36.1	29.7	45.4	55.4	38.6	46.8	66.9	35.4	37.1	41.4
Baby post-natal check within 24 hours	15.0	18.1	15.2	31.8	20.7	19.5	27.7	30.8	4.5	21.5	19.7	5.4	9.0	18.3
Measles immunization	90.3	90.5	96.8	95.2	95.6	85.9	94.9	75.3	88.5	91.7	95.3	79.2	85.4	88.5
DPT3 immunization	97.0	79.2	93.7	98.1	97.6	91.6	96.2	83.0	87.8	93.6	96.1	82.0	92.0	91.3
All-antigen* immunization	86.9	71.7	88.0	93.0	94.9	82.8	92.4	69.6	81.1	88.8	90.1	74.0	81.0	83.7
Vitamin A	75.7	49.2	59.8	80.6	83.4	41.2	89.2	46.0	64.1	72.4	70.8	36.5	65.4	63.6
**Maternal interventions**														
Skilled birth attendance	64.5	77.5	96.4	84.6	73.5	60.7	86.4	55.4	73.1	61.4	80.8	40.1	42.9	67.1
Antenatal visits	93.4	95.5	98.7	99.6	97.1	95.2	99.3	92.4	96.0	94.8	98.7	88.3	92.5	95.1
Family planning	12.9	16.5	32.2	8.4	8.7	8.8	16.8	12.0	28.3	9.0	13.9	7.8	10.0	14.0
Intermittent preventive treatment in pregnancy	36.4	43.8	32.3	52.2	46.9	47.2	51.3	32.3	29.6	40.0	53.2	19.9	58.1	39.2
**Household’s interventions**														
Improved sanitation	24.3	33.2	76.9	15.0	22.4	16.8	11.1	5.1	27.5	25.7	39.7	9.2	8.2	22.5
Improved drinking water	62.5	89.6	94.6	87.3	86.9	65.0	83.7	65.2	76.4	64.3	93.4	61.6	47.6	73.2
Household ownership of ITNs	73.3	81.5	72.7	62.7	52.8	74.0	62.8	83.2	63.3	98.1	95.4	81.5	71.2	74.3
**U5MR (per 1000)**	117	106	85	81	93	101	110	172	101	153	151	197	223	128

*: include BCG, Polio3, DPT3, and measles immunization

Among the child health interventions, those related to immunization had coverage above 80%, the required level for the universal health coverage [17]. Baby post-natal check 24 hours after birth and exclusive breastfeeding had the weakest coverage; 18% and 7%, respectively.

**Fig 2:**
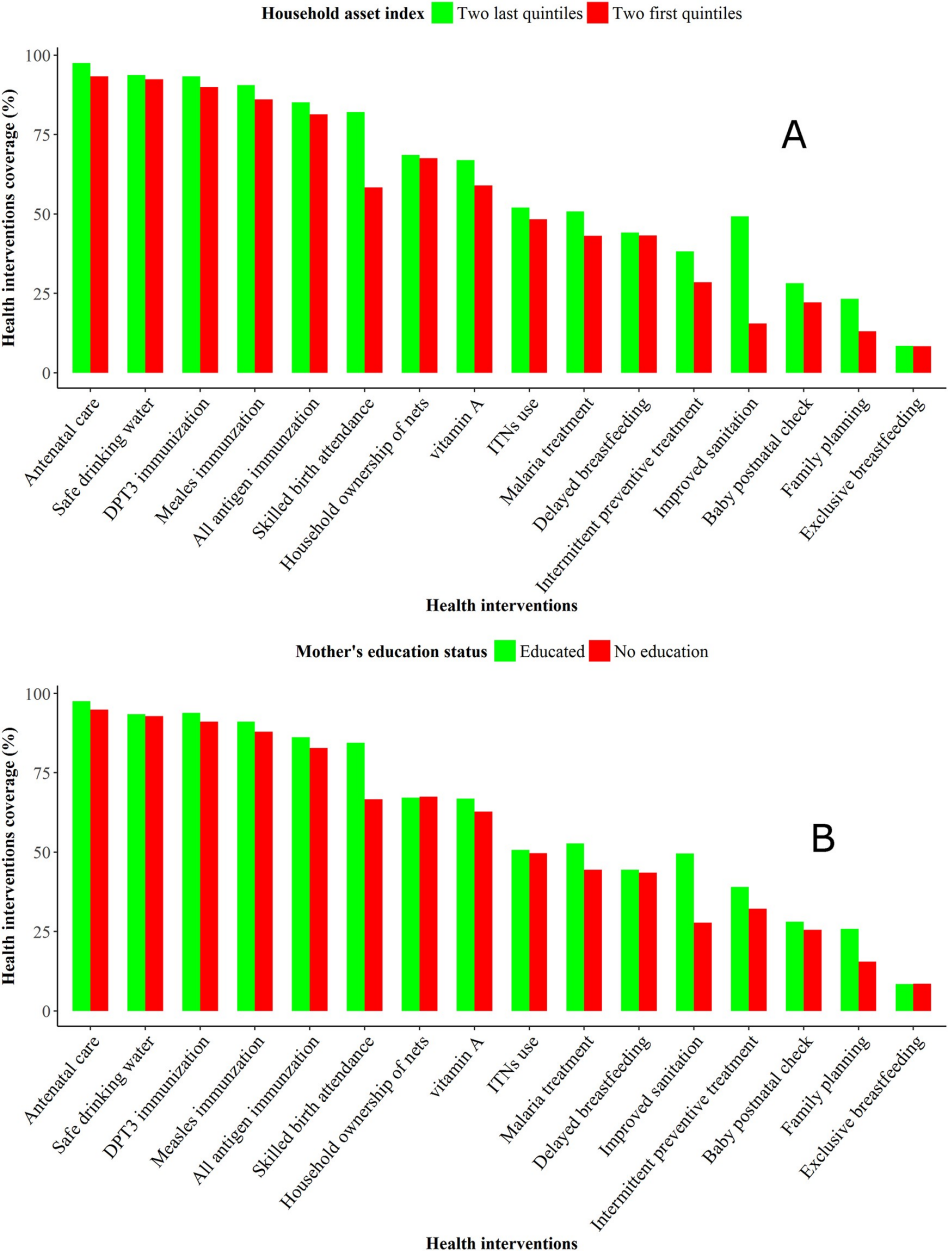
Frequency distribution of the coverage of child health intervention by household asset index (A) and by mother’s education level (B) in Burkina Faso based on DHS 2010.

The coverage of maternal health interventions related to family planning, intermittent preventive treatment of malaria during pregnancy, and antenatal care visit were 14%, 39% and 95%, respectively. Household-based interventions for safe drinking water and ownership of at least one insecticide-treated nets (ITNs) were covering each around 73%. The proportion of households in the country with access to improved sanitation was low; around 20%. The distribution of the health interventions within the 13 regions showed strong heterogeneity. In general, the Sahel, Sud-Ouest, Est and the Centre-Est were the regions with the lowest coverage of most interventions. Geographical disparities were observed in socio-economic proxies, such as the household asset index and the mother’s education level. Fig 2 indicates that the wealthiest and the most educated tended to have high coverage of child health interventions.

**Fig 3:**
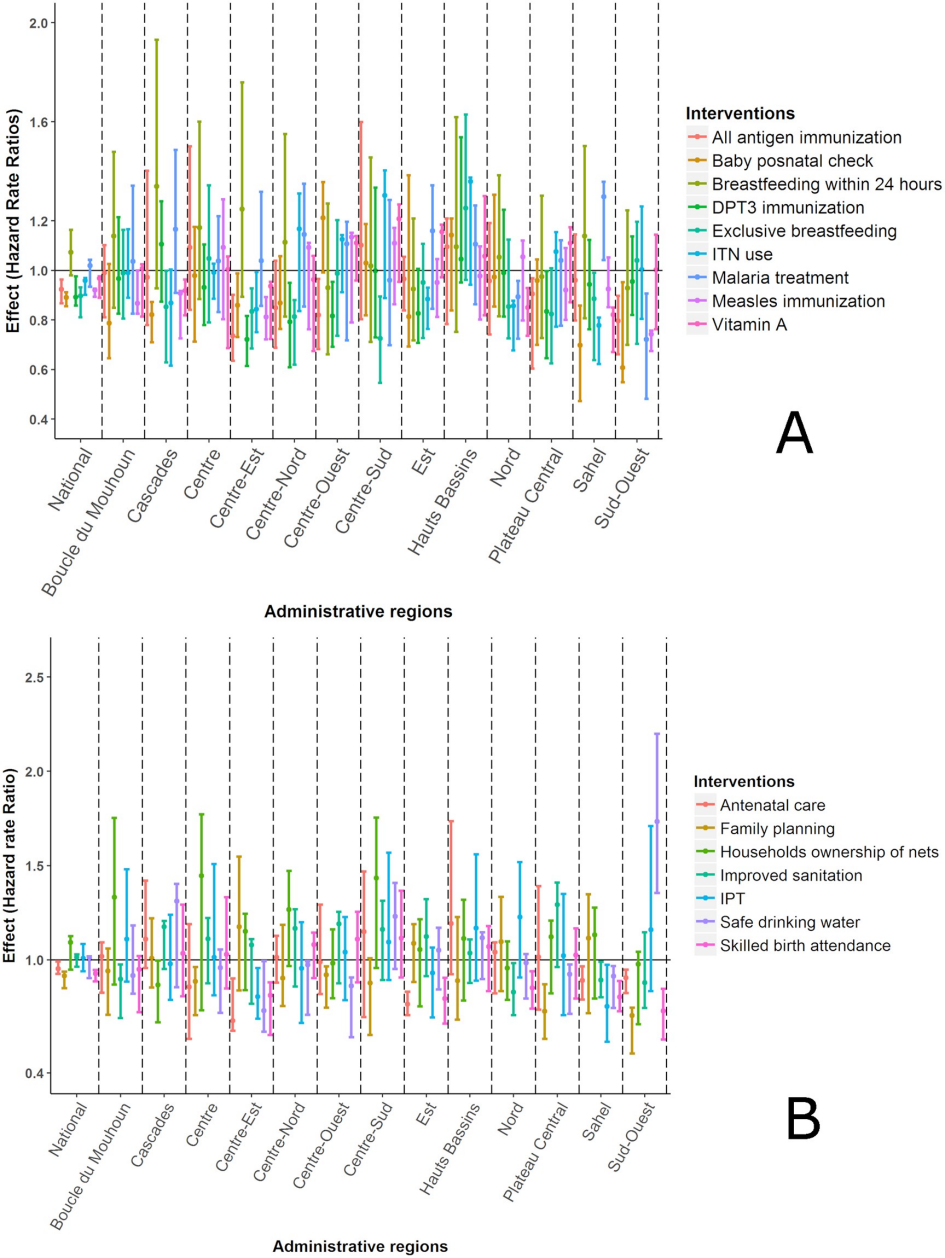
Hazard rate ratios (posterior median and 95% BCI) of child (A) maternal and household (B) health interventions estimated by Bayesian geostatistical Weibull proportional hazards models with spatially varying regression coefficients for the intervention coverage covariates. DHS 2010, Burkina Faso. The horizontal line corresponds to a HRR equal to one.

Results of the bivariate survival analysis in Table 1 indicate that all child, maternal, and household socio-demographic covariates (except sex) were associated with child survival. Tables 3 and 4 show the hazard rate ratios (HRR) of child, mother, and household-specific interventions, estimated by Bayesian geostatistical models, adjusted for socio-economic and climatic covariates. HRR are also provided graphically in Fig 3 and as maps in S3 and S4 Figs.

**Table 3:** Estimates (posterior median and 95% Bayesian credible intervals) of the association between child health interventions at national and sub-national levels and U5MR obtained by Bayesian geostatistical Weibull proportional hazards models with spatially varying regression coefficients for the intervention coverage covariates.

	All antigen Immunization	DPT3 immunization	Measles immunization	ITN use by under five	Malaria treatment	Breastfeeding within 24 hours	Exclusive breastfeeding	Baby post-natal check within 24 hours	Vitamin A
	Hazard rate ratio (95% BCI)	Hazard rate ratio (95% BCI)	Hazard rate ratio (95% BCI)	Hazard rate ratio (95% BCI)	Hazard rate ratio (95% BCI)	Hazard rate ratio (95% BCI)	Hazard rate ratio (95% BCI)	Hazard rate ratio (95% BCI)	Hazard rate ratio (95% BCI)
**Geographical scale**									
**National**	0·92 (0·87–0·96)*	0·89 (0·86–0·98)*	0·91 (0·89–0·95)*	0·95 (0·90–0·97)*	1·02 (0·93–1·05)	1·07 (0·98–1·16)	0·90 (0·81–0·93)*	0·89 (0·86–0·92)*	0·94 (0·89–0·98)*
**Regions**									
**Boucle du Mouhoun**	0·99 (0·81–1·10)	0·97 (0·83–1·22)	0·87 (0·82–1·00)	0·99 (0·89–1·17)	1·04 (0·83–1·34)	1·14 (0·85–1·48)	0·99 (0·81–1·16)	0·79 (0·65–1·03)	1·01 (0·81–1·03)
**Cascades**	0·97 (0·78–1·40)	1·11 (0·87–1·28)	0·91 (0·73–0·92)*	0·87 (0·61–1·00)	1·17 (0·91–1·49)	1·34 (0·93–1·93)	0·85 (0·63–1·00)	0·82 (0·71–0·87)*	0·92 (0·82–0·96)*
**Centre**	1·09 (0·84–1·50)	0·93 (0·78–1·11)	1·09 (0·80–1·29)	0·99 (0·89–1·03)	1·04 (0·83–1·22)	1·17 (0·88–1·60)	1·05 (0·79–1·34)	0·98 (0·71–1·18)	1·00 (0·69–1·06)
**Centre-Est**	0·74 (0·64–0·90)*	0·72 (0·61–0·82)*	0·81 (0·72–0·89)*	0·84 (0·75–0·99)*	1·04(0·86–1·32)	1·25 (0·89–1·76)	0·83 (0·68–0·93)*	0·86 (0·73–0·99)*	0·94 (0·72–0·96)*
**Centre-Nord**	0·85 (0·69–1·04)	0·79 (0·61–0·95)*	1·09 (0·76–1·11)	1·17 (0·84–1·31)	1·15 (0·85–1·35)	1·11 (0·81–1·55)	0·81 (0·62–0·88)*	0·87 (0·76–1·06)	0·96 (0·67–1·06)
**Centre-Ouest**	0·82 (0·68–0·97)*	0·82 (0·69–0·95)*	1·14 (0·79–1·15)	1·13 (0·91–1·14)	1·11 (0·72–1·20)	0·93 (0·66–1·27)	0·99 (0·74–1·20)	1·21 (0·99–1·36)	1·11 (0·96–1·14)
**Centre-Sud**	1·10 (0·80–1·60)	1·00 (0·73–1·33)	1·11 (0·86–1·17)	1·30 (0·89–1·40)	0·96 (0·70–1·28)	1·02 (0·71–1·46)	0·73 (0·55–0·90)*	1·03 (0·82–1·19)	1·21 (0·95–1·27)
**Est**	0·97 (0·84–1·05)	0·83 (0·71–1·01)	0·95 (0·81–1·05)	0·88 (0·73–0·93)*	1·16 (0·84–1·34)	0·93 (0·72–1·21)	0·95 (0·73–1·11)	0·81 (0·69–1·38)	1·16 (0·97–1·19)
**Hauts Bassins**	1·10 (0·78–1·21)	1·05 (0·95–1·54)	0·98 (0·80–1·10)	1·36 (0·94–1·37)	1·11 (0·86–1·26	1·10 (0·75–1·62)	1·25 (0·96–1·63)	1·14 (0·84–1·21)	1·06 (0·82–1·30)
**Nord**	0·96 (0·74–1·19)	0·99 (0·81–1·24)	1·06 (0·80–1·12)	0·86 (0·68–0·89)	0·89 (0·72–0·96)*	1·05 (0·82–1·38)	0·85 (0·73–1·12)	0·97 (0·85–1·31)	0·85 (0·74–0·93)*
**Plateau Central**	0·91 (0·60–0·97)*	0·83 (0·65–1·00)	0·92 (0·80–1·09)	1·08 (0·77–1·16)	1·04 (0·78–1·12)	0·98 (0·73–1·30)	0·82 (0·63–1·01)	0·96 (0·70–1·04)	1·11 (0·87–1·17)
**Sahel**	0·96 (0·80–1·15)	0·94 (0·76–1·12)	0·93 (0·85–1·05)	0·78 (0·62–0·81)*	1·30 (1·04–1·36)	1·14 (0·81–1·50)	0·89 (0·64–0·99)*	0·70 (0·47–0·86)*	0·82 (0·67–0·85)*
**Sud-Ouest**	0·80 (0·66–0·90)*	0·96 (0·82–1·14)	0·74 (0·68–0·76)*	1·00 (0·80–1·26)	0·72 (0·48–0·91)*	0·93 (0·70–1·24)	1·04 (0·70–1·20)	0·61 (0·55–0·95)*	1·00 (0·76–1·14)
**Spatial parameters**	Median (95% BCI)	Median (95% BCI)	Median (95% BCI)	Median (95% BCI)	Median (95% BCI)	Median (95% BCI)	Median (95% BCI)	Median (95% BCI)	Median (95% BCI)
**Range (km)**	17·7 (15·0–59·6)	34·5 (14·3–39·3)	27·6 (19·7–37·0)	37·6 (29·0–47·4)	49·3 (22·6–71·4)	56·0 (8·2–69·5)	39·0 (20·1–64·7)	29·9 (9v6-66·0)	36·4 (8·2–42·5)
**Spatial variance**	0·37 (0·32–0·41)	0·16 (0·14–0·23)	0·16 (0·12–0·19)	0·15 (0·13–0·23)	0·16 (0·12–0·23)	0·19 (0v11-0·33)	0·13 (0·10–0·16)	0·15 (0·13–0·17)	0·16 (0·12–0·34)
**Variance of spatially varying effect**	0·56 (0·36–0·63)	0·17 (0·11–0·20)	0·27 (0·15–0·47)	0·34 (0·20–0·48)	0·33 (0·25–0·57)	0·23 (0·11–0·56)	0·19 (0·12–0·41)	0·31 (0·15–0·37)	0·14 (0·11–0·29)

* Statistically important association.

**Table 4:** Estimates (posterior median and 95% Bayesian credible intervals) of the association between maternal and household health interventions at national and sub-national levels and U5MR obtained by Bayesian geostatistical Weibull proportional hazards models with spatially varying regression coefficients for the intervention coverage covariates.

	Skill birth attendance	Antenatal visit	Family planning	IPT	Improved drink water	Improved sanitation	Household ownership of nets
	Hazard rate ratio (95% BCI)	Hazard rate ratio (95% BCI)	Hazard rate ratio (95% BCI)	Hazard rate ratio (95% BCI)	Hazard rate ratio (95% BCI)	Hazard rate ratio (95% BCI)	Hazard rate ratio (95% BCI)
**Geographical scale**							
**National**	0·93 (0·88–0·96)*	0·95 (0·92–0·98)*	0·91 (0·85–0·94)*	1·01 (0·94–1·08)	1·01 (0·90–1·03)	1·00 (0·96–1·04)	1·09 (0·95–1·13)
**Regions**							
Boucle du Mouhoun	0·95 (0·72–1·02)	1·02 (0·82–1·09)	0·94 (0·71–1·06)	1·11 (0·88–1·48)	0·92 (0·82–1·18)	0·90 (0·69–0·97)*	1·33 (0·87–1·75)
Cascades	1·03 (0·81–1·29)	1·11 (0·96–1·42))	1·01 (0·85–1·22)	0·98 (0·79–1·24)	1·31 (0·85–1·40)	1·18 (0·95–1·21)	0·87 (0·67–1·00)
Centre	1·03 (0·85–1·33)	0·86 (0·58–1·19)	0·89 (0·71–0·96)*	1·01 (0·81–1·51)	0·96 (0·72–1·05)	1·11 (0·88–1·22)	1·44 (0·73–1·77)
Centre-Est	0·81 (0·60–0·88)*	0·68 (0·62–0·90)*	1·17 (0·84–1·55)	0·80 (0·69–0·96)*	0·73 (0·62–0·99)*	1·08 (0·77–1·11)	1·15 (0·84–1·24)
Centre-Nord	1·08 (0·90–1·14)	1·01 (0·88–1·13)	0·90 (0·75–1·19)	0·96 (0·66–1·20)	0·96 (0·71–0·99)*	1·17 (0·86–1·27)	1·27 (0·97–1·47)
Centre-Ouest	1·11 (0·88–1·25)	0·99 (0·82–1·29)	0·92 (0·75–0·96)	1·04 (0·79–1·23)	0·86 (0·59–0·91)*	1·19 (0·87–1·25)	0·98 (0·80–1·16)
Centre-Sud	1·12 (0·91–1·37)	1·15 (0·70–1·47)	0·88 (0·60–1·01)	1·09 (0·89–1·57)	1·23 (0·95–1·41)	1·16 (0·89–1·31)	1·43 (0·96–1·75
Est	0·79 (0·66–0·91)*	0·76 (0·71–0·83)*	1·09 (0·88–1·19)	0·93 (0·69–1·06)	1·05 (0·84–1·17)	1·12 (0·91–1·32)	1·05 (0·75–1·22)
Hauts Bassins	1·07 (0·83–1·18)	1·19 (0·92–1·73)	0·89 (0·68–1·23)	1·17 (0·89–1·56)	1·12 (0·90–1·15)	1·04 (0·88–1·11)	1·11 (0·78–1·32)
Nord	0·85 (0·74–0·94)*	1·04 (0·82–1·09)	1·10 (0·83–1·33)	1·23 (0·91–1·52)	0·98 (0·79–1·03)	0·83 (0·71–0·98)*	0·96 (0·79–1·10)
Plateau Central	1·02 (0·79–1·17)	1·01 (0·79–1·39)	0·73 (0·58–0·87)*	1·02 (0·71–1·35)	0·92 (0·71–0·97)*	1·29 (0·96–1·41)	1·12 (0·82–1·21)
Sahel	0·80 (0·73–0·89)*	0·89 (0·79–0·97)*	1·12 (0·72–1·35)	0·75 (0·57–0·97)*	0·91 (0·74–0·97)*	0·89 (0·80–0·98)*	1·13 (0·79–1·28)
Sud-Ouest	0·73 (0·58–0·85)*	0·90 (0·82 0·95)*	0·70 (0·50–0·75)*	1·16 (0·83–1·71)	1·73 (1·35–3·20)	0·88 (0·75–1·15)	0·98 (0·66–1·04)
**Spatial parameters**	Median (95% BCI)	Median (95% BCI)	Median (95% BCI)	Median (95% BCI)	Median (95% BCI)	Median (95% BCI)	Median (95% BCI)
Range (km)	21·0 (38·1–53·5)	31·2 (23·9–50·3)	18·3 (30·0–68·4)	53·3 (42·2–71·1)	58·9 (33·9–68·9)	37·4 (15·4–70·1)	37·7 (12·4–69·6)
Spatial variance	0·13 (0·13–0·16)	0·15 (0·13–0·20)	0·17 (0·12–0·23)	0·16 (0·11–0·19)	0·15 (0·11–0·18)	0·14 (0·10–0·17)	0·16 (0·11–0·22)
Variance of spatially varying **effect**	0·18 (0·11–0·43)	0·23 (0·16–0·37)	0·21 (0·18–0·98)	0·22 (0·11–0·45)	0·33 (0·22–0·53)	0·19 (0·15–0·40)	0·28 (0·13–0·39)

* Statistically important association.

Breastfeeding within 24 hours after birth and household ownership of ITNs did not have an important association neither at national nor at regional level. At national level, DPT3 immunization and baby post-natal check within 24 hours were associated with a reduction of U5MR (HRR = 0.89, 95% BCI: 0.86–0.98 and HRR = 0.89, 95% BCI: 0.86–0.92, respectively).

There was a considerable variation in the magnitude of the association between the interventions and U5MR within the country. The number of interventions by region associated with a reduction of U5MR ranged from zero to 11 with a median number of three. The region, with the highest number of statistically important interventions were Centre-Est, Sahel, and Sud-Ouest having 11, eight, and seven interventions, respectively. A second group of regions is composed by Cascades, Plateau Central, Nord, Centre-Ouest, Centre-Nord, and Est with three to four interventions associated with child survival. The remaining regions had at most one intervention with a statistically important regression coefficient. No important intervention was identified in the region of Hauts Bassins. The combination of interventions with statistically important coefficients varied by region.

Four out of 13 regions had child-specific interventions with protective effects: all-antigen immunization (Centre-Est, Centre-Ouest, Plateau Central, and Sud-Ouest), exclusive breastfeeding (Centre-Est, Centre-Nord, Centre-Sud, and Sahel), vitamin A supplementation (Cascades, Centre-Est, Nord, and Sahel) and baby post-natal check (Cascades, Centre-Est, Sahel, and Sud-Ouest). Use of ITNs (Centre-Est, Est, and Sahel), measles (Cascades, Centre-Est and Sud-Ouest) and DPT3 immunization (Centre-Est, Centre-Nord, and Centre-Ouest) were associated with U5MR in three regions. Intermittent preventive treatment of malaria in pregnant women was not associated with mortality hazard at national level; however, it was associated with mortality hazard in the regions of Centre-Est and Sahel. Among all child interventions at regional level, baby post-natal check showed the strongest negative association with U5MR in the Sud-Ouest region (HRR = 0.61, 95% BCI: 0.55–0.95). Skilled birth delivery, antenatal care attendance, and family planning are maternal interventions with statistically important coefficients at both national and regional levels. Antenatal care had the highest association with U5MR in Centre-Est (HRR = 0.68, 95% BCI: 0.62–0.90). At national level, none of the interventions related to household was associated with child survival. However, improved drinking water is associated with a reduction in U5MR in five regions (Centre-Est, Centre-Nord, Centre-Ouest, Plateau Central, and Sahel) while improved sanitation in three (Boucle du Mouhoun, Nord, and Sahel) (Fig 3B).

Malaria is one of the main causes of high U5MR in Burkina Faso. Use of ITNs and treatment with any antimalarial showed a protective effect on U5M in three (i.e. Centre-Est, Est, and Sahel) and two regions (i.e. Nord and, Sud-Ouest), respectively.

Association between U5M with socio-demographic characteristics remained the same across the health interventions. That is, male, born at home, birth order higher than five, first birth younger than 19 years, lack of education, mother’s age less than 19 or above 35 years, and poorer household were associated with increased mortality (S3 and S4 Tables).

The models with spatially varying coefficients for IPT and improved drinking water have the highest estimates (i.e. posterior median) of their range parameters suggesting that the spatial correlation in their residuals extend over longer area compared to that in the rest of the models. This implies a weaker spatial correlation structure for the corresponding interventions. However, the credible intervals of parameter estimates across the different health interventions are overlapping; indicating that there are no statistically important differences in the spatial correlation of the intervention coverage indicators. The same result is also observed in the variance parameter of the Gaussian process estimated by the models.

## Discussion

This is the first study to assess geographical variation in the association of child, maternal and household health interventions with child survival in Burkina Faso at regional level, taking into account socio-demographics characteristics and climatic disparities. The geographical distribution of the coverage of the health interventions showed considerable heterogeneity. Interventions with the highest coverage (> 80%) are those related to child immunization and antenatal care visits. Skilled birth attendance, improved drinking water, and vitamin A supplementation had coverage of 60–80% at country level. The promotion of the above mentioned health interventions have history of several decades. Conversely, interventions with national coverage of less than 40% are those whose implementation was strengthened only the year 2000. These include treatment of malaria with ACTs, exclusive breastfeeding, and early breastfeeding after childbirth.

In our analysis, the child and maternal socio-demographic factors associated with child survival (place of residency, place of delivery, number of live birth, mother’s education level, age at first birth, and age group) are similar to those reported by several authors [18,19]. Our findings showed that boys under-five years old have higher mortality hazard than girls as some studies have found in developing countries [20]. The mother’s characteristics (number of live birth, education level, age at first birth, and age group) are interrelated with the household socio-economic status. In African settings, the poorest exhibit highest child mortality because poverty influences their health seeking behaviour. Women from poorer households are most often less educated, less autonomous, and make less use of maternal and child care services [21]. This is highlighted in Fig 2. Socio-economic status is associated with failure to complete immunization, which is an effective child intervention [22]. The Ministry of Health has addressed the financial barriers by subsidizing childbirth and new-born care in 2006 and removed completely the users’ fees for children under-5 years old in 2016. As a result, the use of health services by the mothers has been increased [23]. The high proportion of home delivery in Burkina Faso can be explained by the high proportions of poorest households and of women not educated or living in remote rural areas. As known, home delivery exposes to asphyxia and neonatal sepsis. Several studies reported that women delivering at young age are at higher risk of preterm birth and complicated delivery [24,25]. These are major causes of neonatal mortality which accounts for 44% of under-five mortality [3,25]. Multiple parity (more than five live births) is another risk factor and must be taken into account during antenatal care visit. Multiple parity leads to weak reconstitution of the mother’s nutritional stock, and hence children with higher birth order are prone to low birth weights which impact negatively their survival. Climatic and environmental factors are linked to child malnutrition, family’s income and the development of water-borne diseases in sub-Saharan Africa [26,27].

Burkina Faso has two main seasons; wet and dry. Malaria and water-borne diseases are prominent during the wet season. Acute respiratory infections and meningitis are more prevalent during the dry season. Low precipitation may be protective in the Sud-Ouest region of the country, which receives most rain, but might be associated with increased mortality in the dry north [26] Rainfall is protective in our analyses. We also found a positive association between distance to water bodies and mortality hazard. Similar findings have been reported with regard the spatial distribution of malaria risk in the country [28] A possible explanation could be that people living in close proximity to open surface water bodies are more aware about the risks and then protect themselves.

The interventions assessed in the current study are almost those whose implementation have been regularly monitored at country level to evaluate progress towards the attainment of MDGs [14] They are effective at national and subnational level except the household ownership of net and breastfeeding within 24 hours after birth which did not show any impact on child mortality hazard at subnational level.

The variation of the association between the health interventions and U5MR in Burkina Faso may be explained by factors related to health system performance (such as health workers density, quality of care, accessibility of health facilities, availability of drugs and supplies …), variations in the coverage of interventions and of the climatic and environmental differences across the country. Centre-Est, Sahel and Sud-Ouest are the regions with more than seven interventions with protective effect on child mortality. U5MR is low in Centre-Est but high in the Sahel and Sud-Ouest. Geographically, the three regions belong to the three climatic areas of the country. The Sahel is the driest region and is part of the Sahelian zone; the Centre-Est belongs to the intermediate Sudano-sahelian zone, while the Sud-Ouest belongs to the Sudanian part with the highest rainfall. Furthermore, in 2010, the Sahel and the Sud-Ouest were the poorest regions, while the Centre-Est was one of the richest. In Sahel and Sud-Ouest, even if the interventions are effective, their coverage is below the national average. The financial barrier might play a crucial role in the uptake of health services and care by the population. On the contrary in the wealthiest region of Centre-Est the population makes better use of the health programme.

Cascades, Plateau Central, Nord, Centre-Ouest, Centre-Nord, and Est have three to four interventions with protective effect and the U5MR in these regions is above 100 per 1000 (with exception of the region of Centre-Nord). These regions present weak coverage of various interventions: malaria treatment in Cascades, and Centre-Nord, immunization, skilled birth, and antenatal attendance, improved drinking water and sanitation in Est, family planning in Centre-Nord, and Nord. They belong to the Sudano-sahelian and Sudanian climatic areas. Furthermore, Est, Nord, Centre-Ouest, and Centre-Nord have high proportion of poor households. The health interventions with protective effects are mostly immunization, malaria-related interventions, vitamin A, baby post-natal check, skilled birth, and antenatal attendance. The finding in Plateau Central region is rather surprising. All interventions showed higher coverage than at national level; however the U5MR is among the highest in the country at around 151 per 1000. The interventions with important effects are family planning, improved drinking water and all antigen immunization. Plausible explanations for those results may be a long delay in health seeking behaviour and a low quality of health care in this region.

Centre, Boucle du Mouhoun, and Centre-Sud have only one intervention with protective effect, while there was no intervention associated with U5MR in Hauts-Bassins. The U5MRs in these regions are below the national rate of 128 per 1000. Centre and Hauts Bassins are the wealthiest regions of Burkina Faso with the highest availability of health infrastructures. In Centre region, family planning is the only intervention associated with a reduction in U5MRn. The mortality rate in Hauts-Bassins is 20 per 1000 higher than Centre, and the average coverage of health interventions is higher, however, no health intervention showed a statistically important association with U5MR. Emphasis should be put on ITNs, baby post-natal check within 24 hours, and IPT, which coverage are below the national average. Thus, the reinforcement of these interventions can impact positively child survival. Boucle du Mouhoun and Centre-Sud belong to regions with high proportion of households in the lower wealth index category. Only improved sanitation in Boucle du Mouhoun and exclusive breastfeeding in Centre-Sud are associated with the reduction of U5Mr. These regions have low coverage of improved drinking water (in Boucle du Mouhoun), use of ITNs and improved sanitation (in Centre-Sud).

Child mortality is also influenced by health system related factors, such as the density of health professional, availability of medical products, and quality of health care. It is interesting to note that Centre and Hauts Bassins that have only one or no intervention associated with child survival, respectively, have the highest density of health professionals in the country, although they cover about a quarter of total population [29]. Our results are in the same direction with previous studies that proved association of ACTs with malaria parasitemia at regional level in Centre-Est, Nord and Sahel [28]. Several authors have highlighted the protective effect of net ownership on child mortality, which, however could not be confirmed in the current study [30]. A possible explanation is that the DHS was carried out after a mass distribution of nets, but the mortality estimates are covering the 5-year period prior to the survey and therefore they are not influenced by the household ownership of nets. Breastfeeding within 24 hours after birth was a second health intervention not associated with U5MR. In the literature, early initiation of breastfeeding (less than one hour) is related to reduced neonatal mortality [31]. Our indicator included a delay of 24 hours which may explain the above finding.

A limitation of our study is that the coverage of health interventions is based on self-reporting information, therefore, the measurement may be prone to recall bias. Our analysis assumed that there is no systematic bias recall neither for a given health intervention nor for a given specific region. There are two more limitations which may have led to under-estimation of the association between the intervention coverage and U5MR. In particular, the mortality data are covering the 5-year period prior to the survey. During the latest years the coverage of interventions has increased. The analysis could not take into account the study period because there were no year specific data available. Data were also aggregated at regional level. The few regions in the analysis most likely contributed to a reduced geographical variation in the coverage of interventions. A better approach would have been to further disaggregate the regions according to rural/urban type.

Concluding, the most effective interventions related to U5MR at national level is the DPT3 immunization, followed by the baby post-natal check within 24 hours, exclusive breastfeeding, measles immunization, all antigen immunization, and vitamin A. Low coverage of DPT3 immunization was observed in Cascades, Plateau Central, and Est, of baby post-natal check in Cascades, Centre Est, and Sud-Ouest and of exclusive breastfeeding in Centre, Sud-Ouest, Boucle du Mouhoun, and Hauts Bassins. Skilled birth and antenatal care attendance are the most effective maternal interventions and should be reinforced in the regions of Sahel, Sud-Ouest, Nord, and Est. Child survival can be enhanced by increasing the coverage of improved drinking water in Sahel, Sud-Ouest, and Boucle du Mouhoun and the coverage of improved sanitation in Est, Centre-Sud, Sahel, and Sud-Ouest.

## Supporting information

S1 Table Description of the intervention coverage indicators used in the study. (DOCX)

S2 Table Climatic covariates, sources and spatial and temporal resolution. Data were extracted during the year of 2010.
(DOCX)

S3 Table Hazard rates ratio (posterior median and 95% Bayesian credible intervals) of child, maternal, household socio-demographic and climatic factors used to adjust the association between child health interventions and U5MR. Estimates are obtained by Bayesian geostatistical Weibull proportional hazards model with spatially varying regression coefficients for the intervention coverage covariates.
(DOCX)

S4 Table Hazard rates ratio (posterior median and 95% Bayesian credible intervals) of child, maternal, household socio-demographic and climatic factors used to adjust the association between maternal and household health interventions and U5MR. Estimates are obtained by Bayesian geostatistical Weibull proportional hazards models with spatially varying regression coefficients for the intervention coverage covariates.
(DOCX)

S1 Fig Geographical distribution of the coverage of child health interventions. The coverage are based on quartile cut-offs: (A) all antigen immunization, (B) DPT3 immunization, (C) measles immunization, (D) malaria treatment, (E) ITN use, (F) baby post-natal check, (G) exclusive breastfeeding, (H) breastfeeding within 24 hours, (I) vitamin A supplementation.
(TIF)

S2 Fig Geographical distribution of the coverage of maternal and household health interventions. The coverage are based on quartile cut-offs: (A) skilled birth attendance, (B) skilled antenatal care, (C) family planning, (D) intermittent preventive treatment of malaria in pregnancy, (E) household ownership of bed nets, (F) improved sanitation, (G) improved drinking water.
(TIF)

S3 Fig Spatially varying coefficients of child health interventions on U5MR. Hazard rates ratio estimates (posterior median) obtained by Bayesian geostatistical Weibull proportional hazards model with spatially varying regression coefficients for the intervention coverage covariates. The distribution of the hazard rates ratio are based on quartile cut-offs: (A) all antigen immunization, (B) DPT3 immunization, (C) measles immunization, (D) malaria treatment, (E) ITN use, (F) baby post-natal check, (G) exclusive breastfeeding, (H) breastfeeding within 24 hours, (I) vitamin A supplementation.
(TIF)

S4 Fig Spatially varying coefficients of maternal and household health interventions on U5MR. Hazard rates ratio estimates (posterior median) obtained by Bayesian geostatistical Weibull proportional hazards model with spatially varying regression coefficients for the intervention coverage covariates. The distribution of the hazard rates ratio are based on quartile cut-offs: (A) skilled birth attendance, (B) skilled antenatal care, (C) family planning, (D) intermittent preventive treatment of malaria in pregnancy, (E) household ownership of bed nets, (F) improved sanitation, (G) improved drinking water.
(TIF)
