# Supplementary material for: Geographical variation in the association of child, maternal and household health interventions with under-five mortality in Burkina Faso
Source: PLoS One. 2019 Jul 1;14(7):e0218163. doi: 10.1371/journal.pone.0218163 (PMC6602179; doi:10.1371/journal.pone.0218163)
Supplement: S1 Table — (DOCX) [file pone.0218163.s001.docx]

**Table 5: Description of the intervention coverage indicators used in the study**

| Interventions | Definition |
| --- | --- |
| *Child interventions* |  |
| Use of ITN by under-five years | Proportion of children under 5 years in a household who slept under an ITN the previous night of the survey |
| Malaria treatment | Proportion of children under 5 years in a household who received any antimalarial in the two before the survey |
| Exclusive breastfeeding | Proportion of infants exclusively breastfed during the first six months after birth |
| Breastfeeding within 24 hours | Proportion of infants who started breastfeeding within one day after birth |
| Baby postnatal check within 24 hours | Proportion of infants who have been checked within one day after birth |
| Measles immunization | Proportion of children who received vaccination of measles |
| DPT3 immunization | Proportion of children who received vaccination of DPT3 |
| All antigen immunization | Proportion of children who received vaccination of BCG, Polio3 DPT3 and measles |
| Vitamin A supplementation | Proportion of children receiving vitamin A supplements in the past 6 months |
| *Maternal health interventions* |  |
| Skilled birth attendance | Proportion of births that took place with the assistance of a skilled provider |
| Antenatal care visits | Proportion of pregnant mothers receiving ANC from a skilled provider |
| Family planning | Proportion of married women using any family planning method |
| Intermittent preventive treatment (IPT) | Proportion of women who received intermittent preventive treatment for malaria during pregnancy |
| *Household health interventions* |  |
| Improved sanitation | Proportion of households using improved sanitation facilities |
| Improved drinking water | Proportion of households with improved source of drinking water |
| Household ownership of nets | Proportion of household with at least one net |
