# Supplementary material for: Geographical variation in the association of child, maternal and household health interventions with under-five mortality in Burkina Faso
Source: PLoS One. 2019 Jul 1;14(7):e0218163. doi: 10.1371/journal.pone.0218163 (PMC6602179; doi:10.1371/journal.pone.0218163)
Supplement: S2 Table — Data were extracted during the year of 2010. (DOCX) [file pone.0218163.s002.docx]

**Table 6: Climatic covariates, sources and spatial and temporal resolution. Data were extracted during the year of 2010.**

| Data Type | Source | Spatial resolution | Temporal resolution |
| --- | --- | --- | --- |
| Day/Night Land surface Temperature (LST) | MODIS/Terra^1^ | 1x1 km^2^ | 8 days |
| Normalized Difference Vegetation Index (NDVI) | MODIS/Terra^1^ | 1 x 1 km^2^ | 16 days |
| Land Cover | MODIS/Combined^1^ | 0·5 x 0·5 km^2^ | NA |
| Rainfall | FEWS NET^2^ | 8x8 km^2^ | 10 days |
| Urban rural extent | Global Rural and Urban Mapping project (GRUMP)^3^ | 1 x 1 km^2^ | NA |

^1^Moderate Resolution Imaging Spectroradiometer (MODIS):

^2^Famine Early Warning System (FEWS) Network: https://earlywarning.usgs.gov/

^3^Socioeconomic Data and Applications Center (SEDAC): <http://sedac.ciesin.columbia.edu/data/set/grump-v1-settlement-points>
