# Supplementary material for: Geographical variation in the association of child, maternal and household health interventions with under-five mortality in Burkina Faso
Source: PLoS One. 2019 Jul 1;14(7):e0218163. doi: 10.1371/journal.pone.0218163 (PMC6602179; doi:10.1371/journal.pone.0218163)
Supplement: S3 Table — Estimates are obtained by Bayesian geostatistical Weibull proportional hazards model with spatially varying regression coefficients for the intervention coverage covariates. (DOCX) [file pone.0218163.s003.docx]

**Table 7: Hazard rates ratio (posterior median and 95% Bayesian credible intervals) of child, maternal, household socio-demographic and climatic factors used to adjust the association between child health interventions and U5MR. Estimates are obtained by Bayesian geostatistical Weibull proportional hazards model with spatially varying regression coefficients for the intervention coverage covariates.**

| Covariates |  | All antigen  immunization | DPT3  immunization | Measles  immunization | Malaria treatment | ITN use | Baby postnatal check within 24h | Breastfeeding within 24 hours | | Exclusive breastfeeding | | Vitamin A | |  |
| --- | --- | --- | --- | --- | --- | --- | --- | --- | --- | --- | --- | --- | --- | --- |
| Child characteristics |  | HR (95% BCI)^1^ | HR (95% BCI) | HR (95% BCI) | HR (95% BCI) | HR (95% BCI) | HR (95% BCI) | | HR (95% BCI) | | HR (95% BCI) | | HR (95% BCI) | |
| Sex | *Male* | 1·16 (1·89-1·28) | 1·16 (1·02-1·21) | 1·14 (1·01-1·29) | 1·13 (1·07-1·19) | 1·17 (1·05-1·21) | 1·09 (0·94-1·22) | | 1·16 (1·01-1·18) | | 1·13 (1·04-1·19) | | 1·20 (1·07-1·26) | |
| Place of residence | *Rural* | 1·99 (1·76-2·16) | 4·10 (3·35-4·27) | 1·83 (1·20-2·25) | 1·11 (1·06-1·31) | 1·98 (1·52-2·18) | 1·32 (1·23-1·65) | | 1·36 (1·03-1·57) | | 1·35 (1·23-1·45) | | 1·49 (1·31-1·86) | |
| Place of birth | *Home* | 1·36 (1·19-1·45) | 1·21 (1·16-1·32) | 1·28 (1·14-1·47) | 1·26 (1·18-1·45) | 1·28 (1·12-1·29) | 1·22 (1·12-1·35) | | 1·40 (1·04-1·49) | | 1·32 (1·27-1·40) | | 1·20 (1·19-1·41) | |
| Birth order | *< 5* | 0·66 (0·57-0·71) | 0·66 (0·49-0·70) | 0·66 (0·54-0·79) | 0·61 (0·52-0·73) | 0·64 (0·59-0·77) | 0·64 (0·59-0·80) | | 0·70 (0·58-0·75) | | 0·62 (0·51-1·15) | | 0·73 (0·67-0·73) | |
| Maternal characteristic |  |  |  |  |  |  |  | |  | |  | |  | |
| Age at first birth (years) | *>19* | 0·98 (0·84-1·02) | 0·94 (0·83-1·06) | 0·98 (0·86-1·06) | 0·93 (0·86-1·14) | 0·96 (0·93-1·00) | 0·88 (0·84-0·98) | | 0·95 (0·87-1·00) | | 0·95 (0·92-1·03) | | 1·03 (0·87-1·07) | |
| Number of live births | *>5* | 2·71 (2·50-3·47) | 2·97 (2·50-3·56) | 2·64 (2·25-3·09) | 2·89 (2·47-3·03) | 2·65 (2·00-2·68) | 2·52 (2·37-2·72) | | 2·77 (2·57-2·90) | | 2·81 (1·77-3·18) | | 2·59 (1·83-2·66) | |
| Age group (years) | *<19* | 1·93 (1·79-2·35) | 1·89 (1·59-1·97) | 1·78 (1·55-2·05) | 1·92 (1·56-1·99) | 1·77 (1·58-1·78) | 1·66 (1·53-1·79) | | 1·83 (1·62-1·92) | | 1·86 (1·51-2·13) | | 1·64 (1·31-1·76) | |
|  | *>=35* | 1·61 (1·48-1·79) | 1·64 (1·39-1·70) | 1·57 (1·34-1·85) | 1·67 (1·26-1·84) | 1·51 (1·29-1·56) | 1·46 (1·25-1·58) | | 1·68 (1·58-1·82) | | 1·70 (1·42-1·72) | | 1·51 (1·32-1·69) | |
| Education | *No education* | 1·37 (1·27-1·44) | 1·22 (1·02-1·37) | 1·29 (1·00-1·60) | 1·16 (1·13-1·50) | 1·34 (1·20-1·38) | 1·35 (1·20-1·45) | | 1·22 (1·06-1·47) | | 1·31 (1·07-1·59) | | 1·32 (1·27-1·49) | |
| Household characteristics |  |  |  |  |  |  |  | |  | |  | |  | |
| Asset index | *Middle* | 0·90 (0·80-1·00) | 0·82 (0·74-1·18) | 0·89 (0·76-1·02) | 0·84 (0·75-0·95) | 0·83 (0·74-1·14) | 0·86 (0·72-1·11) | | 0·97 (0·78-1·03) | | 0·83 (0·77-0·88) | | 0·76 (0·74-1·18) | |
|  | *Poorer* | 1·00 (0·88-1·06) | 1·11 (1·04-1·26) | 0·99 (0·87-1·14) | 0·98 (0·89-1·10) | 1·23 (1·17-1·39) | 0·98 (0·88-1·21) | | 1·05 (1·02-1·09) | | 0·94 (0·84-1·08) | | 0·93 (0·82-1·25) | |
| Climatic covariates |  |  |  |  |  |  |  | |  | |  | |  | |
| Land cover type | *Savannah vs grass* | 1·18 (1·10-1·35) | 1·15 (0·92-1·25) | 1·09 (0·92-1·30) | 1·24 (1·05-1·28) | 1·09 (0·99-1·12) | 0·96 (0·92-1·14) | | 1·10 (0·97-1·31) | | 1·34 (1·08-1·36) | | 1·09 (0·92-1·37) | |
| LSTD |  | 1·03 (0·99-1·14) | 0·96 (0·84-1·11) | 0·96 (0·84-1·07) | 1·00 (0·83-1·10) | 0·95 (0·88-1·01) | 1·00 (0·91-1·12) | | 0·94 (0·88-1·05) | | 0·91 (0·84-0·95) | | 1·05 (0·95-1·05) | |
| LSTN |  | 1·77 (1·53-1·94) | 1·05 (0·86-1·53) | 1·43 (1·08-1·59) | 0·35 (0·29-0·65) | 4·44 (2·65-4·94) | 1·06 (0·96-1·23) | | 2·02 (1·80-2·47) | | 1·70 (1·46-1·84) | | 0·71 (0·59-0·78) | |
| NDVI |  | 1·18 (1·06-1·30 | 1·52 (1·25-1·93) | 0·09 (0·07-0·12) | 1·54 (1·45-1·83) | 0·30 (0·21-0·41) | 1·35 (1·23-1·85) | | 0·49 (0·39-0·54) | | 0·66 (0·57-0·72) | | 1·52 (1·24-1·72) | |
| Rainfall |  | 0·91 (0·88-1·01) | 0·94 (0·91-1·04) | 0·92 (0·84-1·02) | 0·91 (0·84-0·93) | 0·97 (0·92-1·00) | 0·92 (0·81-1·03) | | 0·92 (0·88-0·99) | | 0·96 (0·90-1·00) | | 0·98 (0·92-0·98) | |
| Distance to water body |  | 0·99 (0·95-1.03) | 1·05 (0·96-1·13) | 1·04 (0·96-1·12) | 1·07 (0·98-1·08) | 1·05 (0·96-1·11) | 1·08 (1·00-1·09) | | 1·01 (0·97-1·07) | | 1·04 (0·97-1·06) | | 1·07 (0·96-1·09) | |

^1^ HR: Hazard ratio, BCI: Bayesian credible interval; ^2^Bold numbers indicate a statistically important effect
