# Supplementary figures and images for: Geographical variation in the association of child, maternal and household health interventions with under-five mortality in Burkina Faso
Source: PLoS One. 2019 Jul 1;14(7):e0218163. doi: 10.1371/journal.pone.0218163 (PMC6602179; doi:10.1371/journal.pone.0218163)

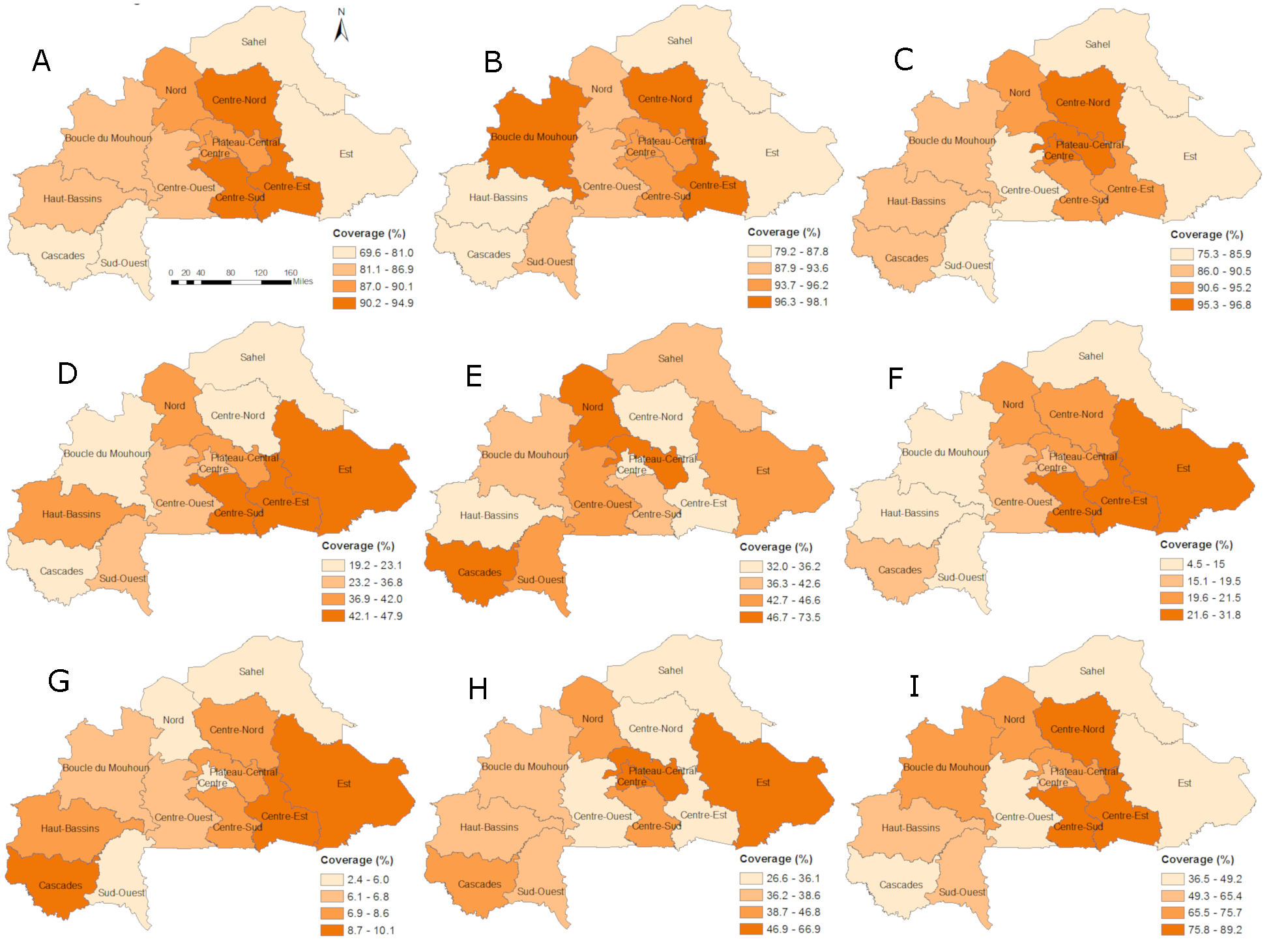

Supplement: S1 Fig — The coverage are based on quartile cut-offs: (A) all antigen immunization, (B) DPT3 immunization, (C) measles immunization, (D) malaria treatment, (E) ITN use, (F) baby post-natal check, (G) exclusive breastfeeding, (H) breastfeeding within 24 hours, (I) vitamin A supplementation. (TIF) [file pone.0218163.s005.tif]

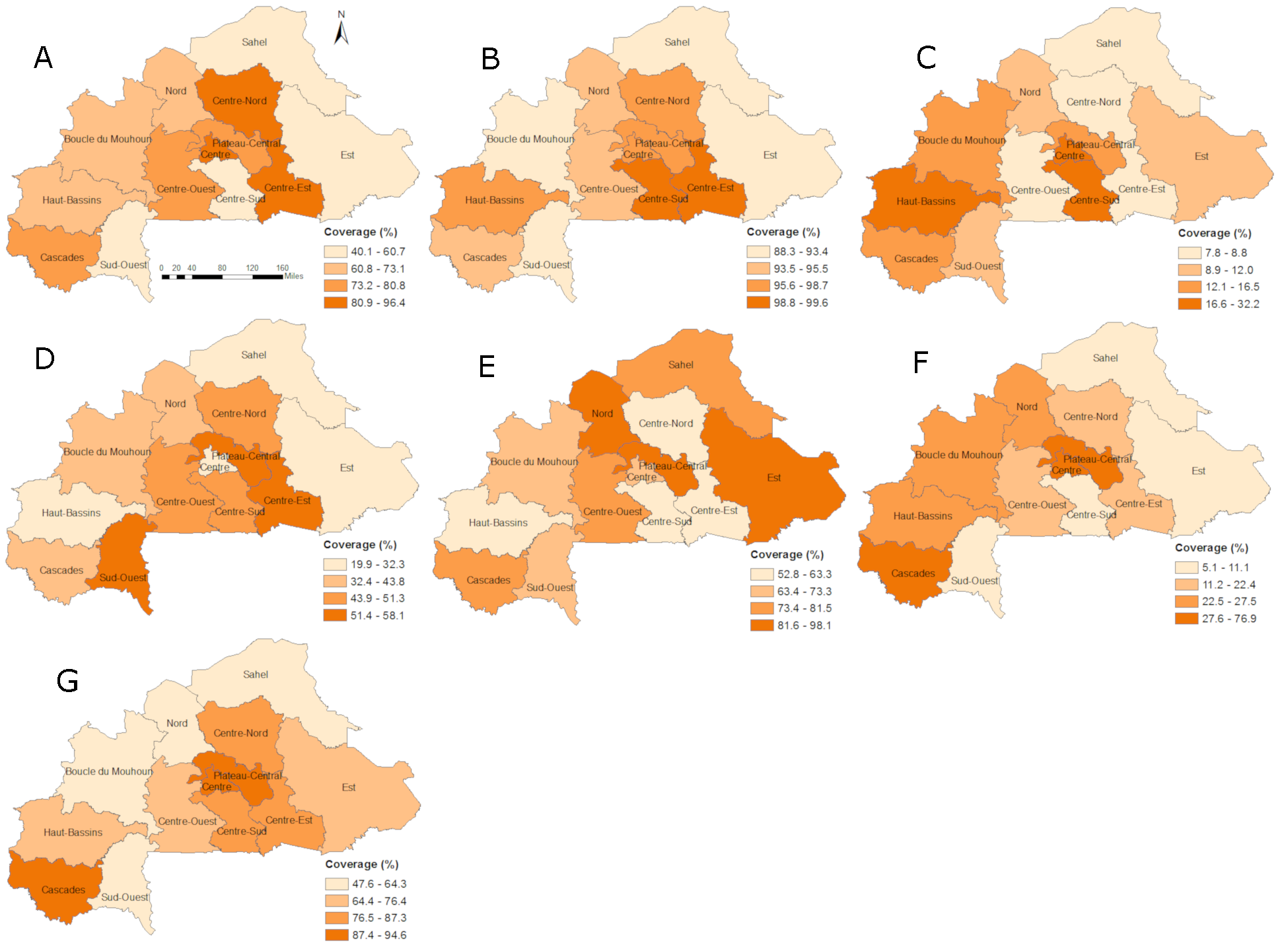

Supplement: S2 Fig — The coverage are based on quartile cut-offs: (A) skilled birth attendance, (B) skilled antenatal care, (C) family planning, (D) intermittent preventive treatment of malaria in pregnancy, (E) household ownership of bed nets, (F) improved sanitation, (G) improved drinking water. (TIF) [file pone.0218163.s006.tif]

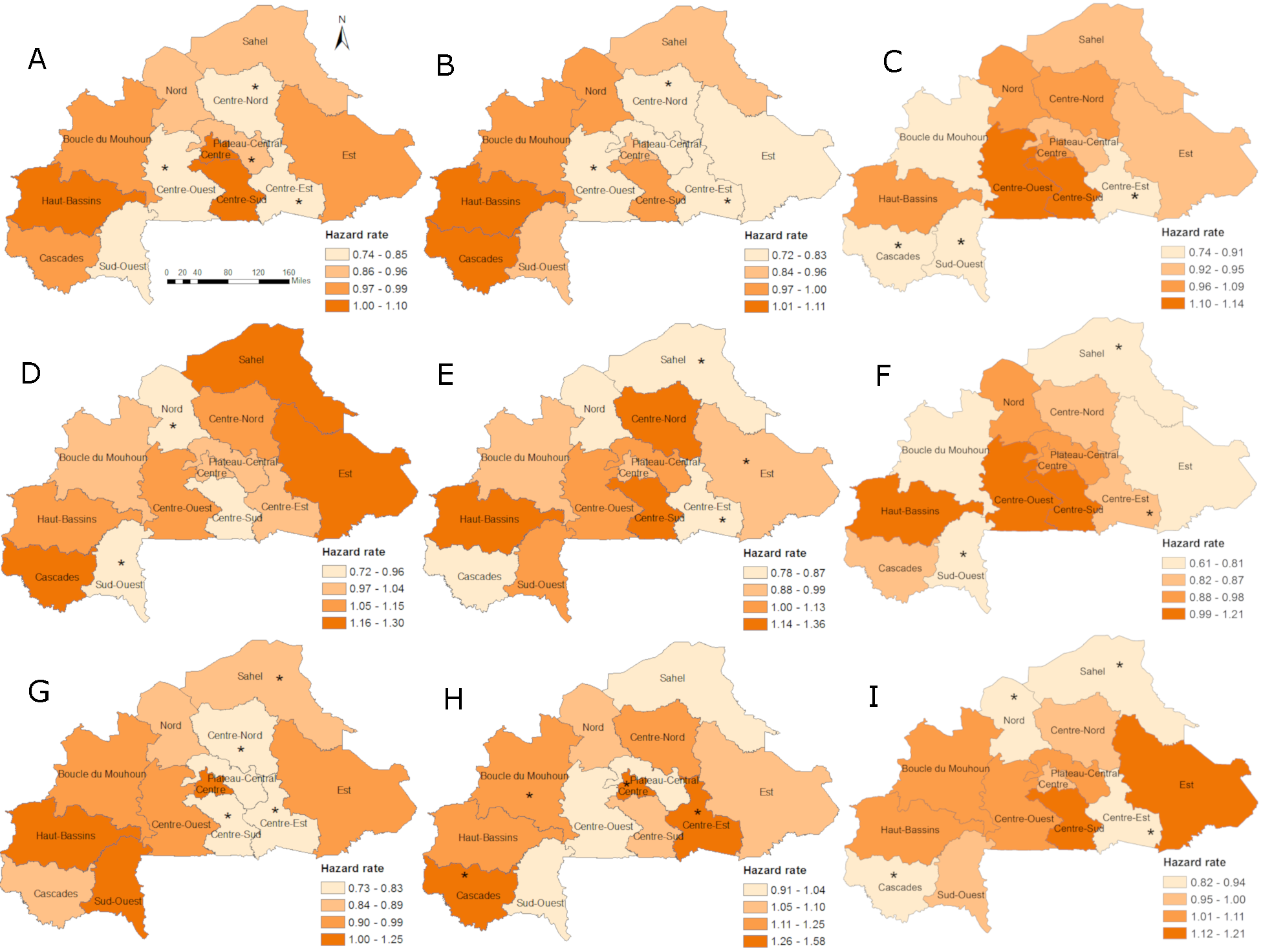

Supplement: S3 Fig — Hazard rates ratio estimates (posterior median) obtained by Bayesian geostatistical Weibull proportional hazards model with spatially varying regression coefficients for the intervention coverage covariates. The distribution of the hazard rates ratio are based on quartile cut-offs: (A) all antigen immunization, (B) DPT3 immunization, (C) measles immunization, (D) malaria treatment, (E) ITN use, (F) baby post-natal check, (G) exclusive breastfeeding, (H) breastfeeding within 24 hours, (I) vitamin A supplementation. (TIF) [file pone.0218163.s007.tif]

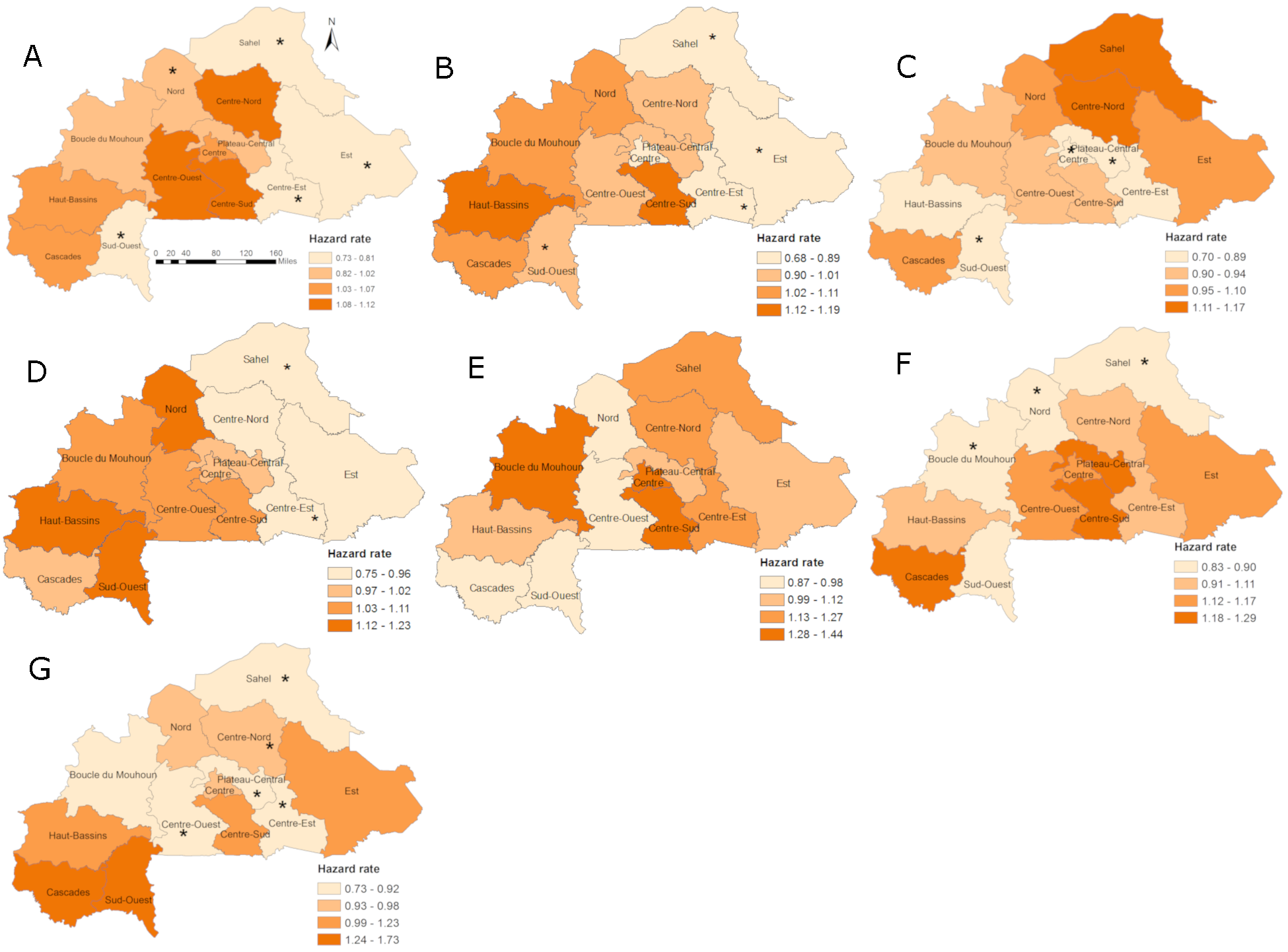

Supplement: S4 Fig — Hazard rates ratio estimates (posterior median) obtained by Bayesian geostatistical Weibull proportional hazards model with spatially varying regression coefficients for the intervention coverage covariates. The distribution of the hazard rates ratio are based on quartile cut-offs: (A) skilled birth attendance, (B) skilled antenatal care, (C) family planning, (D) intermittent preventive treatment of malaria in pregnancy, (E) household ownership of bed nets, (F) improved sanitation, (G) improved drinking water. (TIF) [file pone.0218163.s008.tif]
